# Design of Predictive Tools to Estimate Freshness Index in Farmed Sea Bream (*Sparus aurata*) Stored in Ice

**DOI:** 10.3390/foods9010069

**Published:** 2020-01-08

**Authors:** Juan Calanche, Selene Pedrós, Pedro Roncalés, José Antonio Beltrán

**Affiliations:** 1 Department of Animal Production and Food Science, Meat and Fish Science and Technology Laboratory, Faculty of Veterinary, University of Zaragoza, 50013 Zaragoza, Spain; calanche@unizar.es (J.C.); pedgarsel@gmail.com (S.P.); roncales@unizar.es (P.R.); 2 Department of Food Technology, School of Applied Sciences of the Sea, Nueva Esparta Core, University of Orient, 6301 Nueva Esparta, Venezuela; 3 School of Veterinary Medicine, University College of Dublin, D04 Belfield, Ireland

**Keywords:** partial least square regression, modelling, fish quality tools, shelf life

## Abstract

This research studied sea bream freshness evolution through storage time in ice by determining different quality parameters and sensory profiles. Predictive models for freshness index, storage time, and microbial counts were designed from these data. Physico–chemical parameters were assessed to evaluate the quality of fish; microbial growth was controlled to ensure food safety, and sensory analyses were carried out to characterize quality deterioration. Predictive models were developed and improved with the aim of being used as tools for quality management in the seafood industry. Validation was conducted in order to establish the accuracy of models. There was a good relationship between the physico–chemical and microbiological parameters. Sensory analysis and microbial counts allowed for the establishment of a shelf-life of 10 days, which corresponded to a poor quality (according to the European Community’s system of grading fish for marketing purposes), with a freshness index lower than 50%. Sensory profiles showed that gill and flesh texture were the most vulnerable attributes during storage in ice related to spoilage. The predictive models for the freshness index (%) and ice storage time (h) exhibited an accuracy close to 90% following practical validation.

## 1. Introduction

The common gilthead sea bream (*Sparus aurata*) represents, together with sea bass (*Dicentrarchus labrax*), the two most economically important cultured species in the Mediterranean area [1]. In Europe, the demand for fresh sea bream has increased significantly since the beginning of the century due to its desirable aroma and quality, and its high value has consequently made the farming of the fish a profitable business [2]. The leading producers are Turkey, Greece and Spain, which together covered 87.2% of the European production in 2015 [3]. The main species of farmed fish in the European Union (EU) in 2017 after trout and Atlantic salmon, which tops the list, are seabass and seabream. This last one generated about 95,390 t in 2015, which represented an increase of 14.2% compared to previous year [4].

Fresh and stored in ice are the common commercial practice for most fishes. Keeping fish in ice is one of the most efficient treatments for retarding spoilage. The rate of deterioration during ice storage of fish may vary by species and depends on the concentrations of substrates and metabolites in the tissue, microbial contamination, and conditions of storage after fish catching. The shelf life reflects the susceptibility of fish to deterioration [5]. Regarding this, freshness is an utmost important aspect when assessing the quality of fish. A freshness decrease gives rise to changes in sensory parameters, which have a direct effect upon acceptance by consumers [6]. Changes in fish freshness can be determined by measuring the electrical properties of the fish muscle. Three different instruments are available to measure changes in electrical properties: The Torrymeter^®^ (Distell Industries Ltd., Fauldhouse, West Lothian, UK), the Fishtester VI (IT) (Intellectron International Electronics, Hamburg, Germany), and the RT-Freshness Grader^®^ (RT Rafagnataekni, Reykjavik, Iceland); all of them show a good correlation with fish freshness sensory scores.

Sensory analysis is currently the most important method for freshness evaluation in the fish sector. A recent purpose of the fish sector is to achieve a standardization in sensory analysis by improving the methodologies and training of panels to make sensory evaluation an objective measurement [7]. The physical and biochemical measurements provide information about parameters that are related to fish freshness. However, none of those methods are sufficient to unambiguously determine whether a fish is fresh or not. Time–temperature indicators have been gradually introduced into the wholesale and retail food chain—starting with temperature-sensitive, high-value foods such as fish—with good results, but they are only able to indicate if the cold chain has been maintained satisfactorily.

The shelf life of fresh fish products depends on storage conditions (temperature and atmosphere) and, of course, on initial fish quality. Based on storage trials, empirical shelf life models of the effect of temperature and that of the initial product quality have been suggested [8]. In shelf life studies, some chemical indexes and volatile compounds changes can be monitored and modelled. In fact, effective chemical indexes, mainly based on microbial metabolites, can be independently used—but only after the correlation to spoilage has been well established [9]. However, the models resulting from the combination of several physical, chemical, microbiological and sensory parameters could be much more powerful and versatile [10].

Current applications of predictive models in an industrial context are wide but can be summarized into three groups: (a) product innovation for assessing the rate of microbial proliferation; (b) supporting food safety decisions that need to be made when implementing or running a food manufacturing operation; and (c) incident support to estimate the grade of impact on consumer safety or product quality [11]. Nowadays, predictive modeling in food is a key field of development. The International Committee of Predictive Modeling in Food (ICPMF) was created in 2011; its core mission is to encourage the development of predictive modeling in foods, especially in a distinct range of research topics within the food safety and quality, including modeling quantitative (microbial) risk assessment, predictive modeling in the food chain, quality, and safety management, the modeling of food processes, sampling and plants design [12]. In this context, the main purpose of this research was to study and characterize the spoilage of fresh seabream (*Sparus aurata*) from aquaculture, stored in ice, in order to develop predictive models that are able to estimating quality aspects that could be used as tools for quality management in the seafood industry.

## 2. Materials and Methods

### 2.1. Fish, Storage Conditions and Sampling

A total of seventy-eight sea breams (*Sparus aurata*) from a Spanish fish farm (LES ALFACS, SL, San Carlos de la Rapita, Tarragona) were used in this study. The commercial size was 400–600 g (4/6), and these fish were used in the course of three independent storage trials. The fish were slaughtered by immersion in ice cold water (hypothermia), packed with flaked ice into polystyrene boxes provided with holes for drainage, and delivered to the laboratory within 24 h of harvesting. The fish were held ungutted in a refrigerator set at 1 ± 1 °C, and fresh ice was added as required. Fish stored in ice were analyzed after 0 (≤12 h), 1, 2, 3, 5, 6, 7, 8, 10, 13 and 16 days of storage. On the day of analysis, three randomly chosen fishes were removed from the batch held in ice, and their raw sensory attributes were evaluated; another three fishes were used for physicochemical and microbial determinations. Another two batches with a total of seventeen seabreams—each one and with identical characteristics (4/6) as previously used—were directly provided by another Spanish farm (ACUIGROUP MAREMARE SL., Sagunto, Valencia). These samples were assayed in two different times to get data that were used to validate the accuracy of the designed predictive tools.

### 2.2. Physico–Chemicals Analysis in Seabreams Stored in Ice

#### 2.2.1. Freshness Degree Measured by Torrymeter^®^ (TM)

Changes in the dielectric properties of ice-stored seabream were determined by using a Torrymeter^®^ (Distell Industries Ltd., Glasgow, UK). An average of three measurements, done by using the Torry Freshness Standard Scale-STD (with a 15 meaning fresh and a 1 meaning very spoiled), was obtained for each fish. The instrument base was placed firmly on the fish so that it lied flat against the surface and parallel to the lateral line at a thick, fleshy part of the fish, avoiding the head and belly cavity regions. The electrodes were cleaned between measurements to remove scales and slime, and any remaining ice was cleared from the measuring surface. During the analysis, the internal temperature of seabreams were never less than 0 °C or higher than 1 °C.

#### 2.2.2. Temperature Measurements and pH

An infrared thermometer (Infrared Sensor FR260MV AMR) coupled to a console (ALMENO 2450-1L) that was designed to measure (without contact) surface temperatures (ST) was used. It was placed at a distance of less than 15 cm from skin of the fish, recording and presenting the measures in a digital display. Three measures were made in duplicate, obtaining an average for each point. ST was measured, specifically on the head, trunk and tail of the fish. The internal temperature (IT) was measured by using a puncture electrode. All temperatures were expressed in Celsius degrees (°C). The pH was determined with a digital pH-meter with a puncture electrode (Crison, model PH25). The measurements were performed triplicated in the loin (near of the head) and tail of whole seabreams.

#### 2.2.3. Total Volatile Basic Nitrogen (TVB-N)

Total volatile basic nitrogen (TVB-N) determination was carried out in a Kjeltec unit by direct steam distillation over boric acid, following the protocol described in the European Union (EU) Regulation 2074/2005, Chapter III, “Determination of the Concentration TVBN in Fish and Fish Products” [13].

### 2.3. Microbiological Counts in Fish

Total psychrotrophic viable count (PST), total mesophilic viable count (MVC), *Enterobacteriaceae* (ET) and *Pseudomonas* sp. (PS) were studied in the seabream samples. A piece of fish muscle (10 g) was taken from the dorsal region of each fillet, transferred aseptically into a stomacher bag (Seward Medical, Worthing, UK), mixed with 90 mL of 0.1% peptone water containing 1% NaCl, and homogenized for 60 s with a stomacher (Lab Blender 400, Barcelona, Spain). MVC was determined by pour plate methods in plate count agar (Merck, Darmstadt, Germany) by using conventional dilution procedures. Plates were incubated for 48 h at 30 °C and for 168 h at 10 °C for the MVC and the PST, respectively [14]. For *Enterobacteriaceae*, violet red bile dextrose agar (VRBD, Scharlab, Barcelona, Spain) with a double layer was used, and plates were incubated for 48 h at 30 °C [15]. For the enumeration of *Pseudomonas* sp., samples (0.1 mL) of serial decimal dilutions (0.1% *w*/*v* peptone and 0.85% *w*/*v* NaCl) of fish homogenates were spread on the surface of the appropriate CFC medium (Oxoid code CM 559, complemented with selective supplement SR103 consisting of Cetrimide, Fucidin and Cephaloridine). Petri dishes were incubated at 25 °C for 2 days [16].

### 2.4. Sensory Analyses for Freshness Assessment (Analytical Procedure)

In the first stage, a sensory analysis was carried out to establish the fish spoilage and to design general models. This activity was developed with a panel of ten trained sensory assessors who belong to the Faculty of Veterinary from the *Universidad de Zaragoza*. After that, in the next stage, a practical validation was developed by using another panel with the same number of trained assessor from an aquaculture and biodiversity group (*Universitat Politécnica de Valencia*). Three whole fish were evaluated at each sample time until rejection occurred, applying three score sheets that are frequently used in the seafood industry. In order to achieve a better fit to a normal distribution of data regarding the freshness collected and to get an adequate comparison among them, overall results were weighted for each score sheet used and expressed in %; this was denominated as the “sensory freshness index” (SFI).

Assessors had demonstrated sensory sensitivity in preliminary tests, received considerable training, and were able to make consistent and repeatable sensory assessments of various fish. The panel received prior training with respect to the use of freshness assessment scoresheets according to requirements of International Organization for Standardization (ISO) standards [17]. Along this process, assessors became familiarized with the different scales in order to assess the samples in a more accurate form [18].

#### Sensory Score Sheets Employed

A chart denominated the Freshness Meter Scale of Distell^®^ [19], specially designed for assessing freshness in raw fish, was used. It was modified by taking into account the guidelines of FAO [20] and included four aspects: Odor and description, eyes and appearance, flesh texture, and muscle/belly feature. The scores for each assessed characteristic were summed to give an overall sensory score. This system gave a score of 10 for very fresh fish, while decreasingly lower totals resulted as deteriorated fish. The European economic community’s system of grading fish for marketing purposes [21] was the second score sheet used. In this scale, sensory attributes like the appearance of the skin, eyes, gills and internal organs, surface slime, and the odor and texture of each fish were assessed into four quality grades: E (extra), A (admitted), B (borderline) and non-admitted. However, the categorical data provided by this scheme had to be converted into a decreasing numerical scale from 4 (extra) to 1 (not admitted) in all quality aspects evaluated. The Quality Index Method (QIM), a grading system for estimating the freshness while assessing defects on fresh seabream [22], was the last score system used. This scale considers parameters related to skin, eyes, gills and texture. Once the characteristic of a sensory attribute is determined, it is assigned a demerit score ranging from 1 to 4. Scores for all characteristics were then summed to give an overall sensory grade, designated as quality index. The QIM scale gave a score for absolutely fresh fish (0), while increasingly larger totals (32) resulted as fish deteriorated. Last, the total numbers of defects were added up and expressed as demerit % that was subtracted from 100 (maximum freshness value) to calculate the SFI.

### 2.5. Statistical Analysis

Normality (Kolmogorov–Smirnoff) and homogeneity of variance (Levene test) were confirmed in the data that were obtained from physico–chemical analyses, microbiological counts, and sensory assessments. The statistical analysis of data was done with an ANOVA, and differences among assayed samples were analyzed by using Fisher’s a posteriori test (*p < 0.05*). Likewise, Pearson’s correlations were developed among variables for establish relationships. On the other hand, in sensory analyses, panel performance was evaluated by an ANOVA (products, selected assessor, and interaction product by session) and individual achievement through the coefficient of variation along with central tendency measures according to the standard ISO [23]. Sensory profiles were developed for each sampling time by using the square cosine method from the collected QIM data; this method was useful to identify which descriptors best discriminated a set of different storage time samples and which characteristics of the seabream were important in this sensory study. The software XLSTAT 2016 (Addinsoft^®^, New York, NY, USA) was used for developing the statistical analyses.

#### Design of Predictive Tools and External Validation

In order to develop predictive models based on freshness degree, the Unscramble X V.10.5.1 (CAMO Analytics., Oslo, Norway) was employed to design specific prediction equations by using a partial least-squares regression (PLS-R) analysis and taking into account all studied variables (physico–chemical, microbiology and sensory freshness index) according to a specific methodology indicated by previous research [24,25]. The software was configured as follows: Wide kernel partial least square algorithm due to the fact that it does not handle missing values and is best suited for data with a large number of variables (wide; data set with few objects and many variables); we applied the option “mean center data” and finally developed cross-validation with an uncertainty test and discarded residuals.

Estimations achieved with predictive models that involved microbiological count were validated, by comparison, with those provided by a recognized software for microbiological predictions in food (Seafood Spoilage and Safety Predictor—SSSP) Version 4.0 (2014), which was provided on line for public use by Technical University of Denmark—DTU Aqua [26]. The parameters of this program were: Microbial Spoilage Model, H2S producing Shewanella—specifically “seafood freshness and storage in air” (module I).

## 3. Results and Discussion

### 3.1. Physico–Chemical Parameters in Seabream Stored in Ice

The changes in physico–chemical parameters in seabream stored in ice are shown in Table 1. The electrical conductivity measured by Torrymeter^®^ (TM, West Lothian, UK) showed an initial average value of 14, being 15 the maximum score of freshness, which demonstrated an excellent condition of the fishes.

A gradual and significant (*p* < 0.001) decrease in Torrymeter STD values was observed throughout storage time from day 0 to day 8, when a score of 6.92 was found. In accordance with organoleptic charts for sea bream (*Umbrina canariensis*) [19], this value corresponded to the category of “not admitted” in the UE Scheme [21]. In fact, a value of 6 in the Torry STD scale was indicative of marginal quality of the iced sea bream along its shelf life [27]. These results support the use of the Torrymeter for assessing the loss of freshness of seabream.

The TVB-N values are also shown in Table 1. The initial amount was 5.40 mg/100 g and almost 18 mg/100 g at final sampling time (day 16). Differences among values were significant (*p* < 0.001) through storage time. However, in all cases, the values were lower than those indicated by the legal limit (35 mg/100 g) set for white fish according to regulations [28]. In accordance with this, previous research with seabream reported that the TVB-N concentration fell under the limit of acceptability at the end of the storage period [29]. An amount of 25 mg N_2_ /100 g has been established as value limit in sea bream [30]. Our results are in agreement with those obtained by many researchers [29,30,31,32] who concluded that TVB-N is a poor indicator for the freshness degree of fish. The rise of this parameter is associated with the activity of specific spoilage microorganisms, endogenous enzymes, storage conditions, and hygienic practices [33]. In fact, a high significant (*p* ≤ 0.001) correlation was observed between TVB-N and microbial counts (Table 2).

The values of pH during the storage varied from 6.50 to 6.83. In general, its behavior was fluctuating, coinciding with previously research [30,34,35,36]. The lowest and highest internal temperatures recorded were −0.02 and 0.48 °C, respectively. In the surface of fish, temperature behavior was similar. Both of them showed slight fluctuations but no significant differences. The temperature range of variation for the commercial process in a food processing plant was estimated to vary between 0 and 5.6 °C [35], and the average exposure temperature in the commercial chain is about 0–1 °C during storage and delivery; however, display at the sale points and household storage are the critical stages for temperature increase [9]. Based on the above, temperatures in this study were within the normal variation in the food chain.

### 3.2. Microbial Counts in Seabream Stored in Ice

Microbial counts in seabream through the storage time are shown in Table 1. The MVC and the PST increased over time, with initial values of 3.23 and 3.00 log CFU/g, respectively. These values are lower than initial counts normally found (around 4 log CFU/g) within the known range for this species and farming area [36,37]. After 10 days, the PST (6.89 log CFU*g^−1^) was close to 7 log CFU*g^−1^, which is considered as the maximum level for acceptability of both freshwater and marine fish [38]. However, the MVC (6.42 log CFU/g) exceeded the suggested limit (6 log CFU*g^−1^) provided by regulations [39] after 13 days. These results appear to be similar to those reported in the literature for sea bream that is aerobically stored [31,40,41]. The population of ET was lower than that obtained for other bacteria, but between days 8 and 10, they reached counts *(p* < 0.001) as high as the limit (3 log CFU*g^−1^) of regulations [39]. As is well known, ET counts represent a reliable index of the sanitary quality of fresh and processed foods, providing presumably sufficient protection for consumers [42]. For that reason, a range of 10^2^–10^3^ CFU/g as safety limits for fish have been suggested [43]. High significant correlations (*p* ≤ 0.001) were observed (Table 2) among bacterial counts (PST vs. ET, PST vs. MVC, PST vs. PS, MVC vs. ET, and, finally, MVC vs. PST). Likewise, all microbial counts showed positive significant correlations (*p* < 0.01) with TVB-N values and negative with TM values.

### 3.3. Sensory Profiles and Characterization of Fish Spoilage

The freshness degree for sea bream stored in ice obtained by three sensory methods are shown in Table 1. Very high significant differences (*p* < 0.001) were found in all scales throughout storage time, demonstrating the ability of the panel to discriminate among sampling times. No significant differences were detected between assessors and sessions, ensuring uniformity. Likewise, the coefficients of variation (CV) evaluated for each assessor showed that none exceeded 15%, as recommended by the standard ISO [23].

The sensory characteristics for freshness were excellent according to score sheets (Torry STD and UE) during the first three days and decreased slowly over storage. It is very difficult to find a 100% fresh sample, this being best possible freshness value, in the marketing chain due the time elapsed between capture and transport. For that reason, an SFI ≥ 75% represents a high quality (E), values within the range of 74–50% are demonstrative of a good quality (A); finally, the B and not admitted categories (0–49%) do not achieve minimum requirements, and fish at these levels are therefore considered deficient and prone to rejection. Based on the above, the SFI (EU) index was satisfactory (48.10%) until day 10.

The SFI (%) estimated by sensory assessments (Table 2) showed a high significant correlation (*p* < 0.001) among them. These indices also correlated very well (*p* < 0.01) with microbial counts (MVC, ET, and PST), TVB-N and Torrymeter measurements (TM), as expected. All this indicates that sensory judgements were able to discriminate quality changes and could therefore be used to establish fish freshness in a proper way; these judgements proved, too, to be very useful for predicting the effect of the farms rearing techniques as well as commercial conditions in the food chain [44].

**Figure 1:**
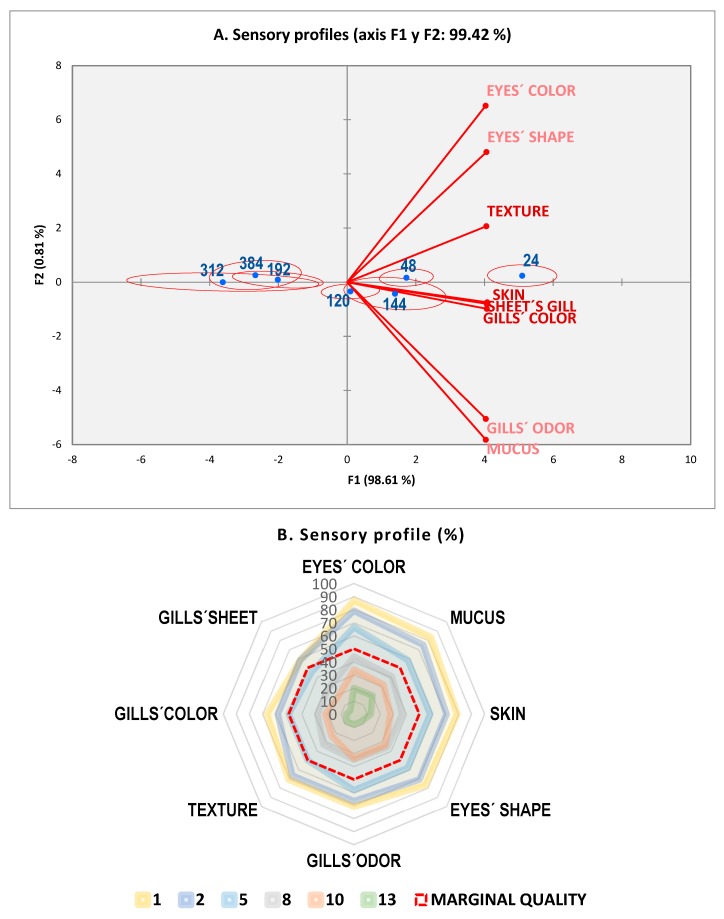
Spoilage over storage time (0 ± 1 °C) by the Quality Index Method (QIM), (**A**) sensory profiles by square cosines coefficients, and (**B**) radial profiles (%) by days.

There was a high correlation (*r*^2^ > 0.90) among the sensory score sheets used (Table 2). Considering the official method (EU), a shelf life (use-by) of 10 days was established that coincided with the limit value for ET’s count. Due to its specificity for assessing sensory characteristics, the QIM was chosen to study fish spoilage. Changes in the sensory profiles of seabreams assessed by the QIM through storage time are shown in Figure 1. Highly significant changes in sensory attributes were established (*p* < 0.01) through storage times (Figure 1A). In general, all descriptors began to receive a poor evaluation from day 8, specially gills that showed discoloration (browning) and sheets that piled up, while texture decreased in firmness; both attributes, together with skin shine, were the worst evaluated and represent key aspects for assessing freshness in seabream. Previous researchers have indicated that earliest and most pronounced negative changes in seabream storage in low temperature (0 °C) slurries were found in gill color (<4 at day 5 of storage) and eye shape (<4 at day 11) [37,45].

As shown in Figure 1A, the plot for the sensory profile demonstrated that most of the variation (98.1%) was due to storage time. This chart allows us to visualize the descriptors and products on the same graph, with a confidence ellipse whose orientation and surface depend on the ratings given by the different assessors [46]. Descriptors (sensory attributes) correlated very well among them and were located in the right side of the biplot. Key aspects for freshness were near day 1. Regarding sampling time, the three groups appeared to be well differentiated (day 1, days 2–6, and days 8–13). Besides this, the ANOVA demonstrated that all attributes were very different (*p* < 0.001) through storage time; sensory profiles of the radial type (Figure 1B) confirmed the sensitivity of key attributes to indicate spoilage evolution.

### 3.4. Global Analysis and Design of Predictive Tools

**Figure 2:**
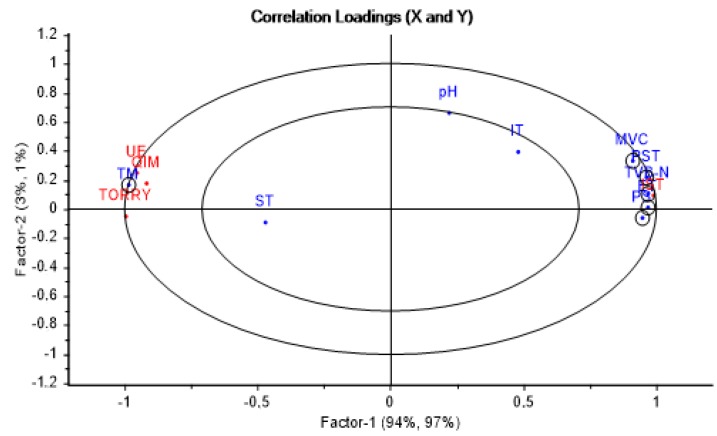
Relation of sensory freshness index (y) and physico–chemical and microbiological parameters (x) by partial least squares (PLS) regression for sea bream during storage in ice. IST: Ice storage time elapsed (h), SFI: Sensory freshness index, TORRY: SFI by Torry scale, QIM: SFI by Quality Index Method, EU: SFI by EU Official method, TM: Torrymeter (STD scale), TVB-N: Total volatile basic nitrogen (mg*100 g^−1^ of fresh fish), pH: PH, ST: Temperature on surface (°C), IT: Internal temperature (°C), MVC: Mesophilic viable count (log CFU/g), ET: *Enterobacteriaceae* (log CFU/g), PST: Total psychotropic count (log CFU/g), and PS: *Pseudomonas* spp. (log CFU/g).

Partial least square regression was carried out with the assessed parameters (Figure 2); sensory responses (SFI) were considered as response variables (Y), while physico–chemical and microbiological parameters were considered as predictor variables (X).

The two first principal factors explained 97% of the correlations found. It can be clearly noted that aspects of freshness (TM and SFI) are located on the left side of the plot, while those of spoilage are on the right (microbial counts and TVB-N). The Hotelling test (T2), a confidence test (p < 0.05), appeared in the plot as two ellipses, which help to check how much of the variance was taken into account when building the model. The outer ellipse represents 100% of the explained variance, while the inner one shows 50%. Based on the above, the individual contribution to PLS-R model of each variable is shown in the plot. The TM value and microbial counts had good weighted regression coefficients; that is why they are enclosed in a circle to highlight them. The value of pH and IT had a moderate weight near the limit of 50% circle, while the ST was negligible. Therefore, important and moderate variables were selected for designing our predictive models.

In essence, a multivariate analysis is a tool to simultaneously find patterns and relationships among several variables. It allows one to predict the effect that a change in one variable will have on the other variables. The development of models by multivariate analyses that combine different standard methods that are capable of providing fast and easy results could represent a new opportunity for estimating quality and commercial aspects such as microbial load, freshness degree and the storage or shelf life of a fish sample.

Predictive models were established for the sensory freshness index (SFI) (%), ice storage time (IST) (h), and mesophilic viable count (log CFU/g) for seabream storage in ice over a period of time ranging from 0 to 13 days. Models were designed based on the combination of all of the studied parameters (physico–chemical, microbiological and sensory responses) that previously demonstrated a god fit and correlations among them. Partial least square regression (PLSR) was used as statistical multivariate analysis that fulfilled the following assumptions: (a) source: Aquaculture; (b) size of the fish: 300–600 g; (c) storage temperature (ST): 0 °C; (d) ST oscillation range: −1.5–3 °C; (e) internal temperature of fish: ≤1 °C; (f) fish condition: Whole; (g) pretreatments: None (unwashed); (h) replacement of ice: Daily; and (i) type of ice: Flake.

General models were obtained from weighted regression coefficients (BOW) graphs created by PLS regression (algorithms) and by taking into account their importance and contribution to the variance of the results. These resulting models were highly complex and cumbersome to apply in commercial practice; for that reason, they were optimized by removing predictive variables with non-significant influence over the studied phenomena and by selecting only those with the best discrimination capacity. This was carried out following an iterative method that resulted in simplified, optimized and validated general equations. The developed tools are described below:

For the SFI, the following equation was designed:

SFI = 4.51 TM + 7.97 pH + 9.88 IT − 7.96 ST − 29.21(1)

where the SFI is the sensory freshness index (%) according to the EU scale, TM is Torrymeter reading (Torry Std. scale), pH is the potential of hydrogen ion, IT is the internal temperature (°C), and ST is the surface temperature (°C). The equation was optimized according to weighted regression coefficients and showed good theoretical validation parameters (slope = 0.97 and *R*-square = 0.95). It allows for the estimation of freshness grade without the use of sensory assessors.

Iced storage time (0 °C), expressed in hours to improve the accuracy of the model estimated in seabream, was established as a function of physical parameters:

IST = 473.43 pH − 10.48 TM − 117.78 IT + 3234.28(2)

where IST is the elapsed ice storage time (h), pH is the potential of hydrogen ion, and IT is the internal temperature. An optimization procedure relying on weighted regression coefficients was made, and good theoretical validation parameters were found (slope = 0.95 and *R*-square = 0.97).

When the Torrymeter^®^ equipment is not available, another equation that involves a traditional physico–chemical analysis could be used:

IST = 19.41 TVB-N − 36.63 pH − 221.43 IT + 137.99(3)

where IST is the ice storage time (h), TVB-N is Total volatile basic nitrogen (mg*100 g^−1^ of fresh fish), pH is the potential of hydrogen ion, and IT is the internal temperature (°C). As in previous cases, the equation has been optimized according to weighted regression coefficients and demonstrated good theoretical validation parameters (slope = 0.95 and *R*-square = 0.97).

Microbial counts are a key criterion for establishing the rejection limit in fresh fish. The current legislation [39] suggests a value of 6 log CFU/g, while a value 7 log CFU/g is considered as the maximum level of acceptability for freshwater and marine fish [37]. Due to the above, an equation based on the SFI and IST was proposed for the estimation of corresponding microbial counts:

MVC = SFI 0.06 + IST 0.020 − 2.64(4)

where MVC is the mesophilic viable count (log CFU/g), the SFI is the sensory freshness index (%) according to EU scale, and IST is the ice storage time (h). A satisfactory validation was found (slope = 0.94 and *R*-square = 0.95).

**Figure 3:**
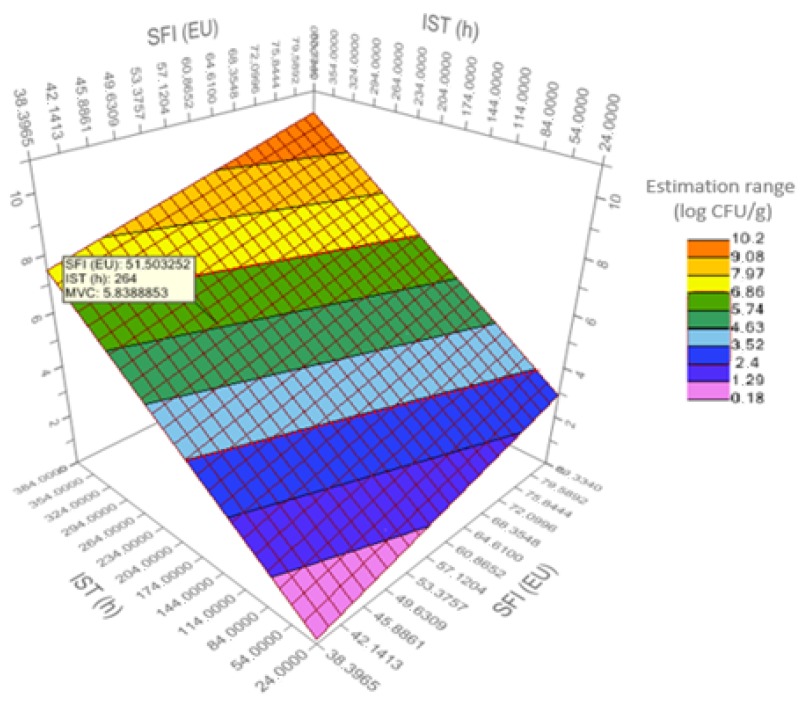
Response surface plot for predictive model of mesophilic viable count (log CFU/g MVC) in sea bream during iced storage. SFI (EU): Sensory freshness index (%) with the European Union official method; and IST (h): Ice storage time of sea bream in hours.

Figure 3 shows the response surface plot (RS) for the bacterial count predictive model (MVC). An RS-plot is used to find the settings of the design variables that give optimal response values, as well as to study the general shape of the response surface fitted by a response surface model or a regression model.

For MVC, the RS-plot established a confidence interval from 0.18 (fresh) to 10.2 log CFU/g (very spoiled), corresponding to a freshness of 90–38%. In food safety management systems, the above equation could be used to set a “critical limit,” i.e., the criteria which separates acceptability from unacceptability in a critical control point [45].

A previous work with seabass demonstrated the benefits that are offered by modeling equations [46], especially for predicting the specific spoilage bacteria (*Shewanella* spp.) that are involved in spoilage, thus contributing to the improvement of food safety control.

### 3.5. Validation for Predictive Tools Developed

The suitability of models developed to estimate the SFI (%), IST (h) and MVC (log CFU/g) was validated. Under normal conditions, the predictive ability of Equations (1)–(4) were tested via a comparison with experimental results. An independent validation experiment was carried out with fish supplied directly from a farm, and this validation was tested by a second sensory panel under the following conditions: A storage temperature of 0 ± 1 °C and high internal temperature of 0–2 °C at different storage times.

**Figure 4:**
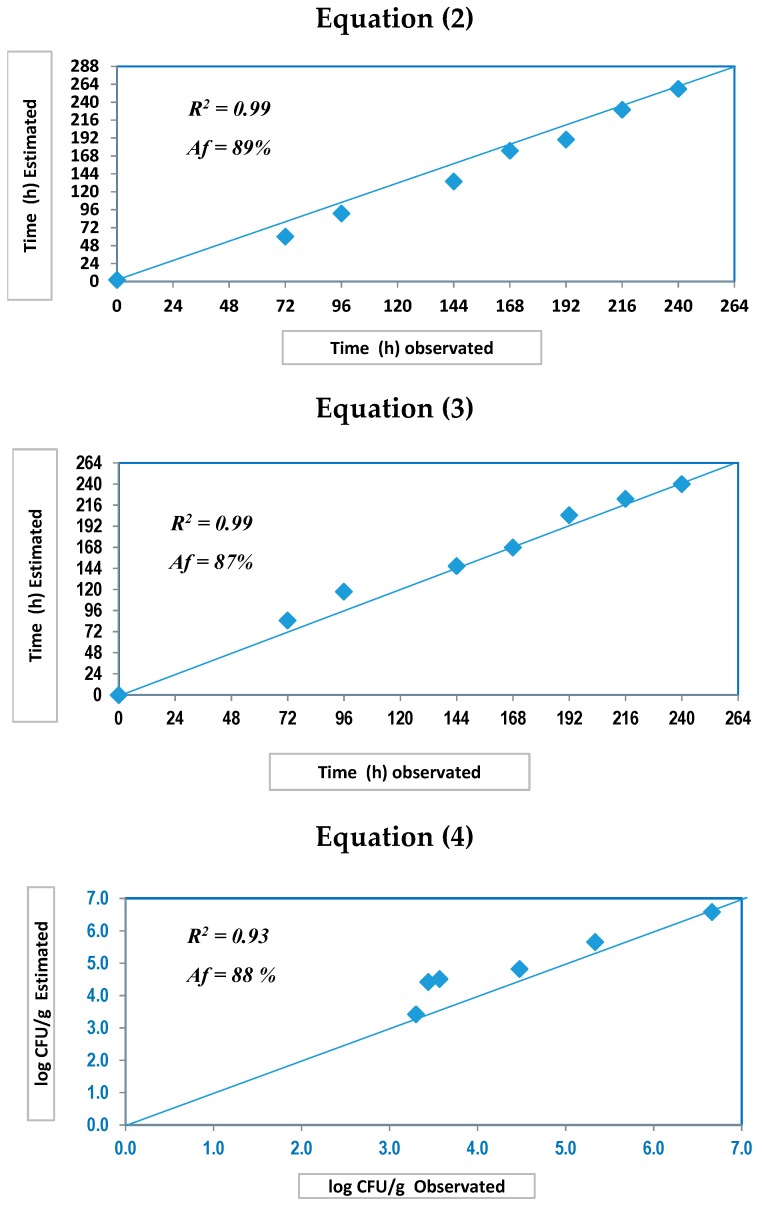
Estimated vs. observed plots for practical validation (Equations (2)–(4)) for seabream over the storage time in ice. Af (%): Accuracy factor.

Figure 4 shows the accuracy that was achieved by the predictive tools. A significantly high 90% was achieved by Equation (1) (SFI). However, accuracy values were different for IST; when taking into account physical methods, Equation (2) showed the best value of the trial with a value of 89%, while Equation (3) (which was based on a combination of physical and chemical parameters) reached a value of 87%. Finally, Equation (4) exhibited a satisfactory performance with an 88% value of accuracy. It has been verified that the proposed tools lose their effectiveness to accurately determine both parameters (SFI and IST) when samples were subjected to temperature abuse in the cold chain (IT > 4 °C). Indeed, a previous study indicated that a quick loss of freshness degree during commercialization could happen following an internal temperature rise up to 5 °C. [36].

**Figure 5:**
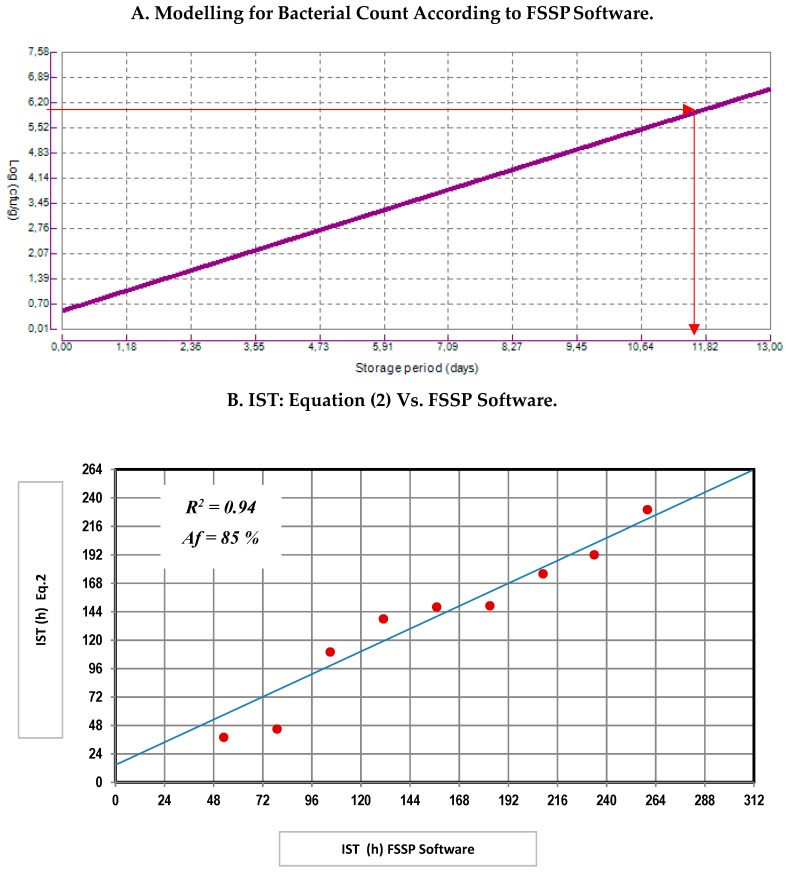
Comparative study between the results for Equation (2) (IST) and bacterial growth according to FSSP software. *IST* (h): Ice storage time of sea bream in hours; *Af* (%): Accuracy factor. (**A**) Modelling for Bacterial Count According to FSSP Software; (**B**) IST: Equation (2) Vs. FSSP Software.

The results of the IST were compared with those provided by the Food Spoilage and Safety Predictor (FSSP) [26]. The FSSP provided a shelf life (use-by) based only on microbiological criteria of 308.64 h (approximately 13 days) (Figure 5A); meanwhile, the IST multivariate tool (Equation (2)) calculated a shelf life of 230 h (10 days) to establish a "not admitted" freshness degree according to EU scale. However, there was an agreement between both models about the required time to achieve the maximum limit suggest to MVC growth, which was allocated as close to 10 days (Figure 5B).

## 4. Conclusions

Predictive models were developed for the freshness index (%), ice storage time (h) and microbial counts; all of these models exhibited an accuracy of close to 90% following practical validation. The findings of this study suggest that the predictive tools designed may be proposed as a valuable alternative to monitor spoilage by assessing freshness as a key aspect of seabream quality. These tools allow for the possibility, too, of avoiding the use of sensory experts and microbiological analysis for estimating freshness degree, time of storage in ice, and microbial load. In a food safety management system, the proposed tools could be employed for quality control and assessment within the cold chain of seabream, as well as for determining freshness loss during logistic and marketing operations. Combining standard traditional methods with these new tools could help decision making and provide additional benefits such as less time consumption, easiness to perform, sensitivity, automation, and the use of non-destructive and non-invasive techniques.

## Figures and Tables

**Table 1:** Quality parameters for iced sea bream over storage time.

Days		TORRY	QIM	EU	TM	TVB-N	pH	ST	IT	PST	MVC	ET	PS
**0**	m	92.24 ^a^	89.08 ^a^	94.76 ^a^	14.00 ^a^	5.40 ^a^	6.50	0.26	0.10	ND	ND	ND	ND
	s	1.34	5.21	1.10	0.28	0.76	0.00	0.11	0.14	ND	ND	ND	ND
**1**	m	84.77 ^a^	85.30 ^a^	83.33 ^b^	13.17 ^a^	8.44 ^ab^	6.50	0.15	0.09	3.00 ^a^	3.23 ^a^	0.43 ^a^	1.16 ^a^
	s	10.25	1.71	13.77	0.06	4.27	0.00	0.13	0.07	0.76	0.53	0.74	0.33
**2**	m	83.72 ^ab^	69.17 ^bc^	80.60 ^b^	12.09 ^a^	8.59 ^ab^	6.65	0.34	0.32	4.01 ^ab^	3.51 ^a^	1.39 ^abc^	1.60 ^a^
	s	4.85	10.03	5.68	0.53	1.76	0.28	0.41	0.35	0.51	0.76	1.32	0.44
**3**	m	80.61 ^ab^	63.28 ^bc^	76.28 ^bc^	12.52 ^a^	10.21 ^bc^	6.41	0.25	−0.04	4.36 ^ab^	3.48 ^a^	1.82 ^abc^	1.98 ^a^
	s	2.50	7.72	6.68	0.10	1.63	0.07	0.43	0.11	0.40	0.46	0.82	0.31
**5**	m	77.15 ^abc^	64.13 ^bc^	69.00 ^c^	10.87 ^abc^	12.82 ^bc^	6.45	0.18	0.01	3.92 ^b^	3.40 ^a^	1.59 ^abcd^	2.55 ^ab^
	s	8.97	10.58	4.48	1.03	1.37	0.16	0.09	0.06	0.94	0.12	1.42	0.42
**6**	m	68.45 ^abc^	52.91 ^cd^	57.30 ^d^	9.55 ^bc^	12.54 ^bcd^	6.43	0.42	−0.02	4.44 ^bc^	3.60 ^a^	1.76 ^abcd^	2.77 ^b^
	s	3.41	12.52	5.29	2.24	1.89	0.17	0.42	0.14	0.63	0.15	1.22	0.33
**7**	m	64.40 ^cde^	42.50 ^d^	50.08 ^de^	7.86 ^bc^	12.85 ^bcd^	6.30	0.31	−0.09	4.97 ^bcd^	4.02 ^ab^	2.68 ^bc^	2.83 ^b^
	s	6.37	8.42	1.97	2.42	2.64	0.00	0.28	0.10	0.72	0.66	0.20	0.25
**8**	m	59.99 ^de^	41.21 ^d^	49.77 ^de^	6.64 ^cd^	13.88 ^cde^	6.44	0.40	0.17	5.43 ^cd^	4.32 ^ab^	3.08 ^cd^	3.01 ^b^
	s	2.44	10.08	2.41	3.15	2.33	0.28	0.07	0.50	1.00	0.63	0.33	0.24
**10**	m	52.62 ^ef^	37.37 ^d^	48.10 ^de^	5.61 ^e^	17.15 ^de^	6.47	0.19	0.23	6.89 ^e^	5.04 ^b^	3.14 ^cde^	2.99 ^b^
	s	6.67	10.37	3.81	3.40	1.45	0.44	0.10	0.36	0.09	1.01	0.25	0.37
**13**	m	42.54 ^f^	32.20 ^e^	44.01 ^e^	4.44 ^e^	17.71 ^e^	6.52	0.02	0.19	8.30 ^e^	6.42 ^c^	4.46 ^e^	3.39 ^b^
	s	12.98	6.62	2.85	3.68	1.81	0.51	0.04	0.50	0.07	0.84	0.68	0.36
**16**	m	36.52 ^f^	33.16 ^e^	38.40 ^e^	2.16 ^e^	17.97 ^e^	6.83	0.07	0.48	8.85 ^e^	7.52 ^d^	4.67 ^e^	3.71 ^c^
	s	19.18	0.00	0.59	2.98	3.27	0.72	0.04	0.71	0.38	0.64	0.02	0.38
		***	***	***	***	***	NS	NS	NS	***	**	***	**

TORRY: Torry scale, QIM: Quality Index Method, EU: Official method, TM: Torrymeter (STD. scale), TVB-N: Total volatile basic nitrogen (mg*100 g^−1^), pH: PH, ST: Surface temperature (°C), IT: Internal temperature (°C), MVC: Mesophilic viable counts (log CFU*g^−1^), ET: *Enterobacteriaceae* (log CFU*g^−1^), PST: Total psychotropic counts (log CFU*g^−1^), and PS: *Pseudomonas* spp. (log CFU*g^−1^). NS: No significant difference, ** = *p* < 0.01, *** = *p* < 0.001. Different letters denote differences with the significance level indicated by the asterisk.

**Table 2:** Pearson correlation coefficients among physico–chemical, microbiological and sensory results for sea bream storage in ice.

Variable Contrasted	Variable Compared	*r* ^2^	Variable Compared	*r* ^2^	Variable Compared	*r* ^2^
**Sensory Analyses (SFI)**						
**TORRY**	EU	0.942 ***	QIM	0.908 ***	TVB-N	−0.953 ***
**TORRY**	TM	0.970 ***	PST/ET	≤−0.965 ***	MVC/PS	≤−0.925 ***
**EU**	QIM	0.951 ***	TM	0.965 ***	TVB-N	−0.908 ***
**EU**	PST	−0.853 ***	MVC	−0.769 **	ET/PS	≤−0.917 ***
**QIM**	TM	0.921 ***	TVB-N	−0.859 ***	PST	−0.853 *
**QIM**	MVC	−0.769 **	ET	−0.930 ***	PS	−0.915 ***
**Physicochemical Parameters**						
**TM**	TVB-N	−0.910 ***	ET	−0.958 ***	PST	−0.920 ***
**TM**	MVC	−0.861 **	PS	−0.911 ***		
**TVB-N**	MVC	0.860 ***	PS	0.962 ***	ET/PST	≥0.901 ***
**PH**	IT	0.881 ***				
**IT**	PST	0.594 *	MVC	0.685 *		
**TM**	TVB-N	−0.761 **	ST	−0.674 *		
**Microbiological Count**						
**PST**	MVC	0.969 ***	ET	0.962 **	PS	0.866 ***
**MVC**	ET	0.904 ***	PS	0.780 **		
**ET**	PS	0.895 ***				
**Storage Time (ST)**						
**ST**	*TORRY*	−0.990 ***	EU	−0.925 ***	QIM	−0.880 ***
**ST**	PST	0.970 ***	ET	0.958 ***	PS/MVC	0.936 ***
**ST**	TM	−0.957 ***	TVB-N	0.960 ***		

TORRY: Torry scale, QIM: Quality Index Method, EU: Official method, TM: Torrymeter (STD scale), TVB-N: Total volatile basic nitrogen (mg*100 g^−1^ of fresh fish), pH: PH, ST: Temperature on surface (°C), IT: Internal temperature (°C), MVC: Mesophilic viable count (log CFU*g^−1^), ET: *Enterobacteriaceae* (log CFU*g^−1^), PST: Total psychrotrophic count (log CFU*g^−1^) and PS *Pseudomonas* spp. (log CFU*g^−1^). Significance level: * = *p* < 0.05, ** = *p* < 0.01, *** = *p* < 0.001.
